# The Addition of Montelukast for the Treatment of Chronic Idiopathic Urticaria

**DOI:** 10.7759/cureus.16137

**Published:** 2021-07-03

**Authors:** Salim Alkeraye, Danah K AlRuhaimi

**Affiliations:** 1 Department of Dermatology, King Khalid University Hospital, Riyadh, SAU

**Keywords:** chronic idiopathic urticaria, antihistamines, leukotriene receptor antagonists, montelukast, singulair

## Abstract

Introduction

Chronic urticaria (CU) is a common disorder that can significantly affect the quality of life. The goal of treatment is complete symptomatic relief. Conventional therapy, with antihistamines, is not always effective in all patients. Leukotrienes are believed to be involved in the pathogenesis of urticaria. Leukotriene receptor antagonists (LTRAs), such as montelukast, have been suggested as useful agents in patients with chronic idiopathic urticaria. Our objective is to document the efficacy of montelukast in our patients.

Materials and methods

Patients who received montelukast were identified from clinic letters. Data including clinical features were collected and analyzed. The main endpoint was adequate disease control.

Results

A total of nine patients who met the inclusion criteria were included in this study. Four patients reported having a good response to montelukast and three patients reported full control of the disease.

Conclusion

These findings suggest that leukotriene antagonists, such as montelukast, are effective as an add-on therapy to anti-histamines and their use in histamine resistant patients is justifiable.

## Introduction

Chronic idiopathic urticaria (CIU) is a common skin condition characterized by the appearance of recurrent wheals and/or angioedema for a duration of at least six weeks without an identifiable trigger. The wheals usually resolve in less than 24 hours. It has been estimated that up to 1% of the general population experiences chronic urticaria (CU) [1]. Although all age groups can be affected the peak incidence is between 20 and 40 years of age and women are more commonly affected than men (2:1). The discomfort experienced with CIU along with the lack of adequate symptoms control can be very frustrating for both patients and health care providers. It has been estimated that the discomfort experienced by those who suffer from CIU can be equal to triple-vessel coronary artery disease [2]. The pathogenesis of CU is complex. Although histamine plays a significant role in CU, prostaglandins and leukotrienes lengthen the inflammatory activity [1].

The international EAACI/GA LEN/EDF/WAO consensus guidelines were published in 2009 for the diagnosis and management of urticaria. The guidelines include a treatment approach, which involves: (A) avoidance measures and (B) pharmacologic therapy. The avoidance method outlines the removal of identifiable causes, avoid physical triggers, and minimize aggravating factors. The second approach is interfering with mast cell mediators with the use of non-sedating second-generation H1-antihistamines such as cetirizine and loratadine, and up-dosing (up to fourfold) if little or no response. However, many patients still remain symptomatic. The addition of H2-antihistamine to conventional H1-antihistamines may be helpful although the evidence of combining H1 and H2 is still poor. Adding sedating H1-antihistamines along with non-sedating antihistamines has shown to be beneficial for some patients; however, studies have shown that sedation is one of the prime barriers in compliance with long-term antihistamines [3].

Montelukast is an active LTRA licensed for the maintenance therapy of asthma and symptoms control of allergic rhinitis. Consensus guidelines suggest the use of LTRA (e.g. montelukast) in patients with CIU resistant to antihistamines. Montelukast 10mg once daily has been used either as monotherapy or in combination with H1 and/or H2-antihistamines to treat various forms of CU, including chronic autoimmune urticaria, delayed-pressure urticaria, cold urticaria, urticaria related to food and CIU [1].

Data from a limited number of systematic clinical trials or case series suggest that some patients with chronic resistant idiopathic urticaria report a good response to leukotriene inhibition [4]. In 2018, the World Allergy Organizations consensus stated that evidence supporting LTRA use was considered weak for strong recommendation [5]. However, we believe that the addition of LTRA, such as montelukast, as an add-on therapy to second-generation H1-antihistamine can significantly improve a patient’s condition. The main objective of this retrospective study is to document the efficacy of montelukast as an added therapy in our patients.

## Materials and methods

The study is an observational retrospective study of patients attending the dermatology clinic at King Khalid University Hospital (KKUH), which is a tertiary care hospital in Riyadh, Saudi Arabia. The study’s proposal was ethically approved by the Institutional Review Board (IRB) of College of Medicine at King Saud University, IRB no. E-21-5784.

Patient selection was based on the following inclusion criteria: age ≥18 years old, confirmed cases of idiopathic CU who are treated with antihistamines in combination with montelukast. The exclusion criterion included patients with identifiable causes of urticaria, patients who required frequent pulsed courses of corticosteroids to control the urticaria, patients who required immunosuppressive agents to control the urticaria, and patients allergic to montelukast. The primary endpoint was adequate to control the disease with the addition of montelukast in CIU resistant to antihistamines. Data were obtained from the electronic medical records at KKUH using a data collection form. Data forms consisted of demographic information and patients' diagnoses and treatment characteristics such as previous medications and their duration and any concurrent use of immunosuppressive treatment. Dose of both antihistamines and montelukast were also included in the forms. The patient's response to the addition of montelukast was measured using the urticaria activity score (UAS).

## Results

A total of nine patients who met the inclusion criteria were identified from clinic letters. There were eight females and one male with ages ranging between 21 and 63. The duration of urticaria ranged between 2 and 10 years. Three out of nine patients were on both second-generation H1-antihistamine and classic (sedating) H1-antihistamines when montelukast 10mg once daily at night was started. The duration of antihistamine use ranged between two months and 10 years (Table 1). Five patients had chronic illnesses including diabetes, hypertension, hypothyroidism, and asthma (Table 1). Seven out of nine patients were better after the initiation of montelukast. Patient number 1 is 63 years old who had been treated with H1-antihistamines (up to fourfolds) for 10 years with minimal response showed full control of her disease with a UAS score of 0 after montelukast was added within a week. She is urticaria free currently and off treatment for three years. Patient number 2 is a 28-year-old female who was suffering from CIU for two years showed complete clinical remission of the disease after two weeks of montelukast use and is off treatment for one year with UAS of 0. Patient number 3 is 30 years old who showed full control within two weeks after montelukast was started she is off treatment for one year with a UAS of 0. Patient number 4 is 21 years old who had poorly controlled urticaria despite the use of multiple antihistamines reported better control of symptoms after the initiation of montelukast, however, not complete resolution. She continued to develop wheals that were troublesome and interfering with her sleep with UAS of 2. She reported drowsiness as a side effect in which she discontinued the drug after one month of use. Patient number 5 is 46 years old with poorly controlled CIU for two years who reported poor control of the symptoms despite the use of montelukast. Patient number 6 is 58 years old with CIU for two years who showed no difference in the disease activity after the initiation of montelukast with UAS of 2. Patient number 7 is a 33-year-old male who reported improvement of his disease after starting montelukast. His UAS is 1. Patient number 8 is a 59-year-old female who reported improvement by almost 60% with a UAS of 1 after starting montelukast. She reported weight gain as a side effect of the medication. Patient number 9 is a 45-year-old female who is being treated with montelukast reported better control of her symptoms with UAS of 1 (Table 2).

**Table 1:** Clinical characteristics with details about the antihistamines used. QID - four times a day; BID - twice a day; TID - three times a day; HS - at bedtime; OD - right eye; DM - diabetes mellitus; HTN - hypertension

Patient No.	Age	Gender	Duration of urticaria	Allergies	Chronic illnesses	Antihistamines	Duration of antihistamines use
1	63	F	10 years	nil	Hypothyroidism	Loratadine 10 mg QID	10 years
2	28	F	2 years	nil	Asthma	Loratadine 10 mg QID	2 years
3	30	F	2 months	nil	None	Loratadine10 mg BID	2 months
4	21	F	4 years	nil	None	Loratadine 10 mg BID Hydroxyzine 25 mg HS	4 years
5	46	F	2 years	nil	None	Loratadine 10 mg TID	2 years
6	58	F	10 years	nil	DM, HTN	Loratadine 10 mg TID	10 months
7	33	M	4 years	nil	None	Loratadine 10 mg BID Hydroxyzine 25 mg HS	4 years
8	59	F	3 years	nil	HTN, Asthma	Loratadine 10 mg BID	3 years
9	45	F	3 years	nil	Hypothyroidism	Loratadine 20 mg TID Hydroxyzine 25 mg HS Chlorpheniramine 4 mg OD	3 years

**Table 2:** Clinical characteristics of patients and response to montelukast. UAS - urticaria activity score; LTRA - leukotriene receptor antagonist; S/E - side effect

Patient No.	Age	Gender	Duration of urticaria (yrs)	UAS prior to the use of montelukast	Duration of antihistamines (yrs)	Response to montelukast	UAS after the use of montelukast	Duration of montelukast use	S/E of LTRA
1	63	F	10 years	2	10	Full control	0	1 week	No
2	28	F	2 years	2	2 years	Full control	0	2 weeks	No
3	30	F	2 months	3	2 months	Full control	0	2 weeks	No
4	21	F	4 years	3	4 years	Better	2	1 month	yes
5	46	F	2 years	3	2 years	No difference	3	2 years and ongoing	no
6	58	F	10 years	2	10 months	No difference	2	10 months and ongoing	no
7	33	M	4 years	2	4 years	Better	1	1 year and ongoing	no
8	59	F	3 years	2	3 years	Better	1	3 years and ongoing	yes
9	45	F	3 years	1	3 years	Better	1	1 year and ongoing	no

## Discussion

Histamine is a key mediator of mast cell degranulation; however, leukotrienes also play important functions. Leukotrienes are potent proinflammatory mediators, and these effects can be blocked by LTRAs. Montelukast is an LTRA that shows a marked selectivity and affinity to the cysteinyl leukotriene receptor type 1. It was first approved for clinical use by the US FDA in 1998 with the brand name Singulair [6].

Few systematic clinical trials have demonstrated that patients with resistant CIU benefit from leukotriene inhibition. Erbagci et al. previously demonstrated that patients treated with montelukast monotherapy had lower UAS as compared to the control group, and the need for rescue therapy with antihistamines was significantly lower [7].

This study shows that most of the patients were middle-aged females. Previous international studies also found an increased incidence of CIU in middle-aged females similar to our study population [2].

The primary effectiveness variable, which is UAS, is a semi-objective method for evaluating the activity/severity of urticaria. The results show that there was an improvement in UAS after the initiation of montelukast and a significant reduction in UAS was evident from the first week of therapy. Moreover, one study showed that patients with CIU treated with Zafirlukast, which is an active LTRA, as an add-on therapy to H1-antihistamines show higher disease control and improvement when compared to the control group treated with only antihistamines [8]. Sakar et al. conducted a double-blind, randomized controlled trial on the use of low dose non-sedating antihistamine in combination with montelukast for the treatment of resistant CIU. They found that patients in the montelukast group showed fewer side effects and improved Dermatology Life Quality Index (DLQI) as compared to the group treated with higher doses of antihistamine monotherapy [3]. To compare these findings to our study, only two out of eight patients reported side effects to montelukast, one being increased day drowsiness and the other is weight gain. 

Akenroye studied the use of montelukast as an add-on to second-generation antihistamines in the patient diagnosed with angioedema predominant urticaria. They found that 92% of patients who received montelukast improved with a high number of complete symptom resolution in the follow-up period ranging from four months to three years [5]. Furthermore, Kosnik and Subic conducted a double-blind crossover study in which they found that the subgroup with more severe disease antihistamine resistance was more likely to benefit from the addition of montelukast [9]. Erbagci observed in a single-blind crossover study that the subgroup taking montelukast required less frequent use of antihistamines than with placebo group [7]. Similarly, our patients reported less frequent use of antihistamines after the initiation of Montelukast and three patients reported complete remission and discontinuation of both antihistamine and montelukast, the longest being off treatment for three years. Study limitations include the relatively small number of patients, which requires further experimental studies to support our findings.

## Conclusions

The LTRA, montelukast, plus anti-H1/H2 therapy was effective in most of our patient population. This case series shows that montelukast can be a successful treatment option for patients with CIU who are not adequately controlled with antihistamines. Moreover, the excellent safety profile without the need for regular blood monitoring is the reason why LTRAs are considered the preferred additional agents in our patient population.
